# The Role of Advanced Parental Age in Reproductive Genetics

**DOI:** 10.1007/s43032-023-01256-2

**Published:** 2023-05-12

**Authors:** Boling Chu, Zhi Liu, Yihong Liu, Hui Jiang

**Affiliations:** 1 Department of Biobank, Suining Central Hospital, Suining, 629000 China; 2 Department of Pathology, Suining Central Hospital, Suining, 629000 China; 3 College of Humanities And Management, Guizhou University of Traditional Chinese Medicine, Guizhou, 550025 China

**Keywords:** Advanced Parental Age, Genetics, Offspring, Neurocognitive, Emotional

## Abstract

The increase of parental reproductive age is a worldwide trend in modern society in recent decades. In general, older parents have a significant impact on reproductive genetics and the health of offspring. In particular, advanced parental age contributes to the increase in the risk of adverse neurodevelopmental outcomes in offspring. However, it is currently under debate how and to what extent the health of future generations was affected by the parental age. In this review, we aimed to (i) provide an overview of the effects of age on the fertility and biology of the reproductive organs of the parents, (ii) highlight the candidate biological mechanisms underlying reproductive genetic alterations, and (iii) discuss the relevance of the effect of parental age on offspring between animal experiment and clinical observation. In addition, we think that the impact of environmental factors on cognitive and emotional development of older offspring will be an interesting direction.

## Introduction

The reproductive age increased over recent decades concomitantly paralleled with increase in social economy and education level in countries. The proportion of primiparous mother aged 30 years or older increased steadily from less than 7% in 1968 to 44% in 2005 in Canada. The percentage of reproductive men increased by 48% at the age of 35–39 and by 51% at the age of 40–44 in USA from 1980 to 2010 [1]. Furthermore, the number of advanced age parturient women has increased more recently in China as the government policy allows the birth of a second child [2]. However, advanced parental age not only increases the risk of adverse pregnancy outcomes, but also associates with poor long-term health outcomes in offspring [3], which could be possibly due to genetic alterations, including chromosomal, telomere, and epigenetic aberrations.

The effects of parental age on offspring’s cognitive and emotional development differed in population-based observational studies. Some reports indicated that advanced maternal age (AMA) positively impact offspring cognitive scores [4, 5]. However, another study found an association between AMA with lower IQ scores in offspring [6]. The difference between the two studies is that the former examined maternal socioeconomic status and parenting environment during delivery, both of which have capacity to predict child academic performance. A review by Jessica et al. suggested that a relationship between a variety of socioeconomic and psychological factors with both offspring outcomes and maternal age has an impact when general patterns of child behavior were taking into consideration [7]. However, unlike women, the effects of advanced paternal age (APA) on offspring are manifested more in the increased risk of developing neuropsychiatric disorders (e.g.: bipolar disorder [8], autism [3], and schizophrenia [9]) in the offspring, as well as phenotypes of mental retardation [6], neurocognitive developmental impairment [10], and impaired sociability [11]. In cognitive and emotional development, Saha et al. found a strong correlation between the father’s reproductive age and the decreased offspring scores [5]. Similarly, Malaspina et al. found an inverse association between the offspring's nonverbal IQ and the father’s age during adolescence [6]. Contrarily, when investigators consider parental socioeconomic status (SES), parental education, and the number of siblings in the family, they found that the relationship between offspring’s intelligence scores and paternal age was increasingly attenuated [12]. The results of the studies based on human behavior are contradictory, all of the above findings can only demonstrate association and not causation. So, it is necessary to understand the genetic mechanisms of the impact of advanced parental on offspring.

## Changes in Ovary (or Testis) Fertility and Biology with Age

### Ovary

Ovaries are one of the most important humans’ reproductive organs. In women, age-related decline of female ovarian function starts at the age of 30 and ends around the age of 50 [13, 14]. The ovary contains 6 to 7 million oocytes during the female fetal period. After maturation, a few oocytes (400–500) are fertile because most oocytes are lost due to apoptosis or programmed cell death [15, 16]. Oocyte loss occurs at the same rate throughout reproduction, with the slope of decline remaining consistent with premenopausal [17]. Consequently, oocyte quality (the inherent ability of the oocyte to resume and complete meiotic maturation, to be fertilized and support preimplantation embryonic development, and to result in the production of a healthy offspring [18]) and ovarian reserve decline with age in women. The age-associated decrease in oocyte number do not fully explain the effect of age on the ovary aging. A decrease in follicle quality in the microenvironment of the oocyte or an increase in aneuploidy further affects fertility.(including reactive oxygen species production, mitochondrial damage, telomere loss, and changes in methylation) [19]. Overall, due to a reduction in oocyte quality and number decline, ovarian aging results in ovarian failure [20].

### Testis

In the male’s reproductive system, age influences the morphology and function of testicles. The mean testicular volume increases from 11 years old, maintains at the age of 30–60, and dropped gradually after 60 years old [21]. The thickness of the tunica propria of the seminiferous tubule’s basement membrane significantly increased while the seminiferous epithelium was reduced during aging. These above changes may lead to a narrowing of seminiferous tubules [22]. Animal experiment found that the age of the male rats was associated with reduced pregnancy rates and offspring mortality when they mated with young female rats [23]. Therefore, the quality of the sperm will decline over time, resulting in a decrease in the sperm cells’ fertility and an increase in preimplantation loss. It is likely that a negative relationship exists between age and daily sperm production, total sperm count, and viability. However, other experiments also showed contrary conclusions. The relationship between male age and sperm concentration is till debated in some research [21, 24]. The female factor (partner age) and the decrease in coital frequency with age might contribute to the influence too. So, the effect of age on male fertility cannot be accurately evaluated. But most studies agree that delayed conception increases with male age [25].

### Changes in Germ Cell Genetics with Parental Age

#### Chromosomal Abnormalities

##### Oocyte

The most prevalent chromosomal aberration found in oocytes is aneuploidy. It is frequently caused by meiosis mis-segregation during meiosis and can be classified into two class: whole-chromosome nondisjunction and precocious chromatid separation [26]. The proportion of aneuploidies increases with AMA, leading to an increased risk of genetic disorders (most notably Down syndrome (trisomy 21), Turner syndrome (Monosomy X), and Edwards syndrome (trisomy 18) [27]. However, not all chromosomes have the same probability of forming triploid or inducing aneuploidy with advancing maternal age [28]. Trisomy 16 is mainly caused by maternal errors during meiosis I, whereas trisomy 18 is primarily caused by maternal faults during meiosis II (~ 70%) and even somewhat by meiosis I error [29–31]. Therefore, chromosomal abnormalities in oocytes tend to occur on specific chromosomes, and these abnormal chromosomes occur at different times during meiosis [28]. A potential explanation for age-related chromosomal changes may be that multiple potential factors interaction contribute to age-related chromosomal damage and mis-segregation (for example: mitochondrial dysfunction, telomere shortening, cohesins dysfunction, and spindle assembly checkpoint (SAC) impairment) [32].

##### Sperm

Chromosome abnormalities in sperm are generally caused by meiotic errors occurring during early spermatogenesis, and these include chromosomal numbers (aneuploidy) and structural (translocations, inversions, duplications) abnormalities, with about a 9% rate of chromosome abnormalities in spermatozoa, 7% of these being structural and 1–2% being numerical [33, 34]. Advanced paternal age increases sperm with X Y aneuploidy, mainly 47, XYY Klinefelter syndrome, and 47, XXY Klinefelter syndrome [35, 36]. Furthermore, the frequency of sperm chromosomal abnormalities in terms of structure has been shown to be strongly related to paternal age. It has been observed that the incidence of structural chromosomal anomalies in spermatozoa is 2.8% in males aged 20–24 and increases to 13.6% in men older than 45 [35]. Nonetheless, there is insufficient evidence that the aforementioned correlation increases the risk of having children with de novo structural chromosomal abnormalities [37]. Interestingly, trisomy 21 aneuploidy is a special case. After adjusting for maternal age and other characteristics, the risk of trisomy 21 was twofold higher among fathers at the age older than 50 compared to those aged 25 to 29. This highlights that trisomy 21 aneuploidy increases with paternal age [38]. Currently, age-related chromosomal changes may be explained by lifelong cell divisions (mitotic and meiotic) during spermatogenesis, which put germ cells at greater risk of recombination errors, chromosomal injury, and gene conversions. Furthermore, chromosomal changes also increase gradually with age due to cumulative exposure to environmental toxins and cell damage.

#### Telomeric Alterations

A telomere is the terminus of a linear chromosome consisting of a tandem repeat of DNA sequence (TTAGGG) and binding proteins that protects its integrity [39, 40]. TTAGGG repetitive sequences are maintained by telomerase, a ribonucleoprotein complex consisting of a template RNA (TERC) and reverse transcriptase subunit (TERT) [41, 42]. However, there may be differences in the dynamics of telomeres across men’s and women’s life spans due to the inherent aging risks on reproduction. Notably, a telomere’s length is highly heritable, and transmission of telomeres across generations can occur either genetically or epigenetically [43]. As a result, aging exhibit different effects on telomere length in germ cell, and further affect the health in their offspring.

##### Maternal

After maturation, the activity of telomerase in oocytes remains low, it only increases during blastocyst formation, suggesting that fetal oogenesis achieves maximum telomere length [44, 45]. This implies that telomere length was “fixed” in the maturing oocyte. However, two “hits” in the germline of women compromise the telomeres, according to the Telomere Theory of Reproductive Aging [46]. The initial “hit” occurs in early development when fast mitotic oogonial divisions shorten telomeres. The second “hit” happens during the prolonged interval between ovulation and meiotic arrest. At this time, oocyte telomeres are shortened even more by reactive species, which are byproducts of cellular metabolism. Oocytes containing shortened telomeres produce fewer chromosomal crossovers, predisposition to aneuploidy, and undergo apoptosis during the preimplantation embryo stage of development. This suggests that the telomere shortening in oocytes has the potential to affect prenatal outcomes.

Moreover, animal study has analyzed the influences of telomere length on the reproduction genetics of female. Over generations, mice lacking telomerase activity (TR − / − mice) experienced progressive telomere shortening. After a few generations, these mice developed telomere-depleted chromosomes and chromosomal abnormalities, as well as female sterility. In addition, a series of events occur before sterility occurs in late generation TR mice, including shrinking litter size, degraded meiotic spindles, chiasmata, cytoplasmic fragmentation, and embryonic arrest [47]. Similar results emerged in human studies. According to a study of women having IVF, IVF for older women resulted in shorter telomeres than for younger women experiencing the same reproductive procedure, and this difference translates to a greater proportion of miscarriages or aneuploid embryos [48]. At least, the current studies demonstrate that abnormal telomere lengths in the oocytes of AMA may contribute to some risks that AMA poses for their offspring.

##### Paternal

Unlike oocytes, the length of telomere in sperm increases with age [49–51]. This may be due to the persistent action of telomerase, which is highly expressed in spermatogonia [51, 52]. There is a positive cumulative effect across generations due to the impact of paternal age at conception on offspring’s telomeres [53]. For example, offspring of APA has longer telomeres and lives longer [54]. This indicates that the effect of paternal age on the telomere length of offspring could provide a mechanism to lengthen the offspring’s life span or to compensate for short telomere length in oocytes [55]. In addition, telomere length was found to be longer in semen from healthy old subjects than young subjects, and children of APA had longer telomeres than the children conceived from young fathers [55].

An animal study analyzed age-related changes in telomere “messages” in sperm. The study found that the regions where age-associated changes(e.g.: DNA methylation) typically took place were enriched in sub-telomeric regions [56]. Importantly, telomeric subregions can escape the large-scale epigenetic reprograming events following fertilization and during early sperm development, highlighting that methylation markers in telomeric subregions may be inherited to offspring [57]. In addition, mutations in telomere subregions can easily lead to mental retardation in offspring. Therefore, a relationship between the intergenerational transmission of telomeres and epigenetic is possible.

#### Alterations in DNA Methylation

##### Maternal

Unlike somatic cells, which have highly methylated and stable DNA, germ cell DNA methylation is dynamic and crucial in the growth and development throughout life (Table 1) [58]. An animal experiment found that germ cells of mice underwent genome-wide demethylation at the primordial stage (the during of primordial germ cells to oocytes before meiosis is the primordial stage). But methylation ceases after oocytes enter meiosis, and only after birth did they undergo re-methylation during the growth of oocytes from primary to secondary follicles [59]. A clinic study examined the methylation patterns of genomic DNA in granulosa cells of young (mean age of 26) and elderly women (mean age of 40). Yu et al. found that with the increase of age, the regions with high methylation degree in young women’s genomes will have higher methylation degree. On the contrary, areas with low methylation will continue to decrease. This highlights a shift in the pattern of DNA methylation towards two poles as people aging. Furthermore, Yu et al. have also assessed the effect of methylation difference on gene expression. The 3397 genes differentially expressed between the two groups, 1809 were downregulated in the elderly group. It is worth noting that 1809 downregulated genes contain genes related to ovarian function. (e.g., anti Mullerian hormone (AMH)) [60]. This suggests that germ cell DNA methylation change with maternal aging.

**Table 1:** DNA methyltransferase, their biological function, and changes in oocytes

Enzymes	Biological function	Expression of DNA methylases during oocyte developmental stages	Changes in oocytes with aging
DNMT1	Methylation maintenance	Secondary follicle stage Zygotic stage and beyond	DNMT’s and methylation in MII oocytes and preimplantation mouse embryos
DNMT2	Methylation of transfer RNA	Undetectable at any stage	
DNMT3A	Involved in de novo methylation processes	Primordial stage	
DNMT3B	Involved in de novo methylation processes	Primary follicle stage	
DNMT3L	Does not methylate DNA, but rather it helps facilitate DNMT3a, and DNMT3b activity	Preimplantation embryos	

In addition to studying the patterns of methylation changes that occurred in oocytes, the researchers also found epigenetic and reproductive outcomes associated with maternal age [61]. Immediately after fertilization, the preimplantation embryos (4-cell, 2-cell, morula, and 8-cell) showed significantly lower DNA methylation levels in old mice (35–40 weeks) than young mice later the blastocysts showed no significant differences in DNA methylation [62]. Similar results appeared in earlier studies. Lopes et al. believed that maternal aging did not affect the methylation of DMRs (differentially methylated regions) at imprinted genes or genome-wide DNA methylation levels in placentas and embryos [63]. Based on current finding, after developing into mid gestation stage, embryos from old mother have normal DNA methylation patterning [64]. Therefore, we speculate that (1) although maternal age can adversely affect overall methylation during oogenesis and preimplantation development, there may be a “threshold” that allows imprinted genes to possess normal methylation and preserve DNA methylation ability to maintain and acquisition; (2) a family of methyltransferases (DNMTs) may play a role in preimplantation; (3) DNA methylation imprints within sperm may provide a compensatory mechanism.

##### Paternal

In recent years, an increasing number of studies suggested that APA had adverse effect on offspring’s health [65, 66]. Neither the causal mechanism nor the mode of inheritance of this paternal age effect has been clearly established. One potential mechanism is the epigenetic mutation in sperm over time. The presence of specific genomic age-related hypermethylation sites in the sperm was suggested in an earlier study by Oakes et al. [67]. Researchers found that DNA methylation changes occur consistently or predictably in more than 140 genes in sperm with aging through human cohort studies [68]. An analysis of semen samples from individuals at age of 20 ~ 73 revealed that three CpG sites showed high association with advanced age [69]. Thus, sperm epigenetic information changes with the father’s age. Importantly, a lot of epigenetic alterations are found in the promoter or regulatory regions that involve in neurological and developmental disorders (e.g.: bipolar disease, schizophrenia, mood disorders, and autism). The study by Milekic et al. found that methylation loss in regions flanking the transcription start site in spermatozoa of APA is preserved in the brains of offspring, and impaired expression of genes involved in developmental [70]. Similarly, Yoshizaki et al. reported that DNA hypomethylation in aged spermatozoa plays a key role in the onset of adult disease in offspring of (APA) [71]. This suggests that DNA methylation changes in the sperm of APA may pose some risks to offspring.

**Fig. 1:**
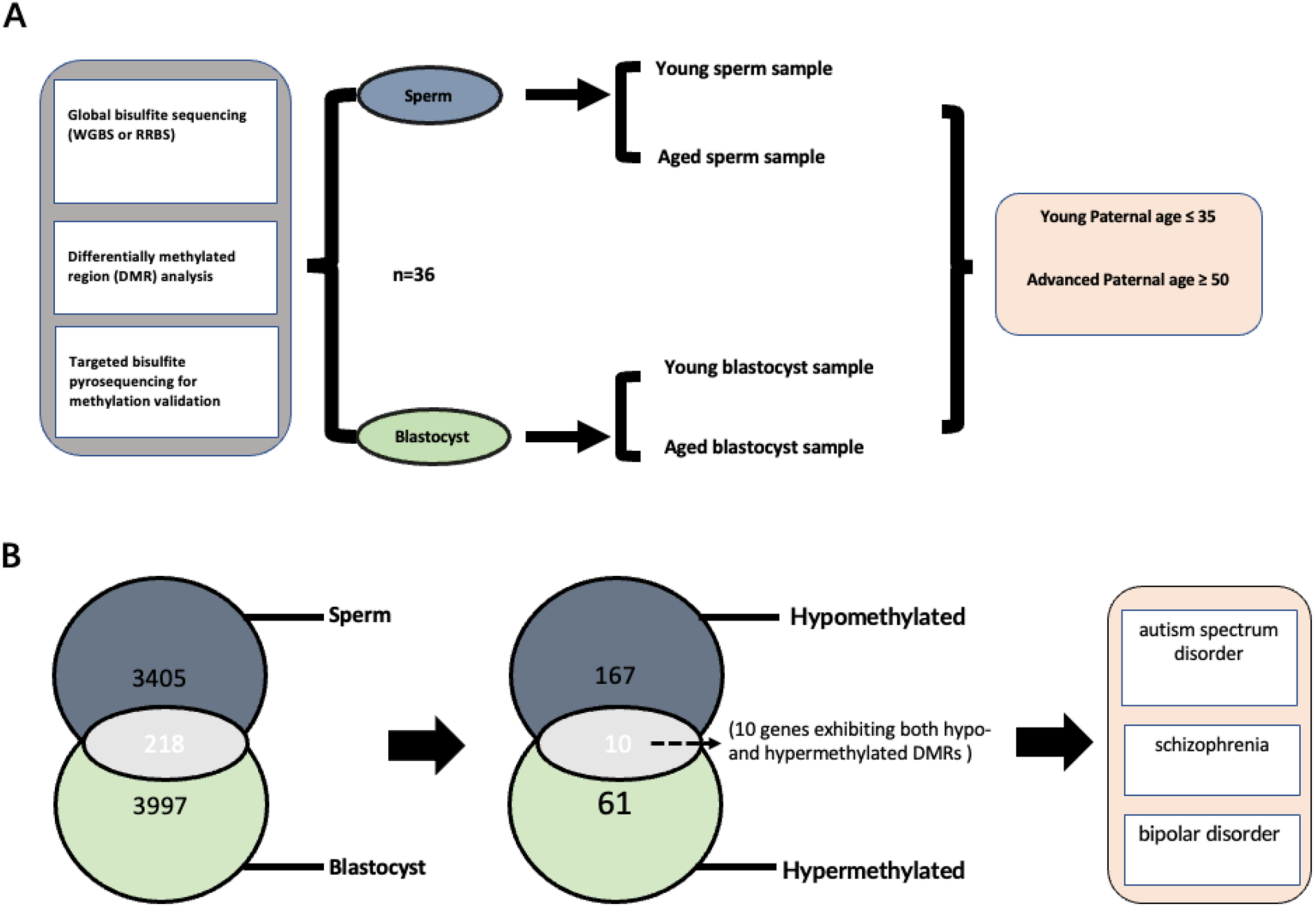
Age-related DNA methylation research on human sperm and blastocysts. **A** Schematic of study design and sperm and blastocyst epigenetic investigations. **B** Schematic of study findings. Significant enrichment of neurodevelopmental genes associated with schizophrenia, autism spectrum disorder, and bipolar disorder was observed in both APA sperm and blastocyst DMRs. Derived from [72]

A recent human study also examined the association between paternal age and neurodevelopmental problems in offspring. In addition to the patterns of DNA methylation changes that occurred in sperm, the study examined methylation changes in blastocyst from APA for the first time. The study design is shown in Fig. 1. Sperm from men with known normal semen parameters and surplus cryopreserved blastocysts from couples undergoing IVF treatment were divided into groups of young and APA. The patterns of global methylation were determined by pyrosequencing, differentially methylated region (DMR) analysis and targeted bisulfite pyrosequencing for the validation of methylation were also performed. In general, a statistical analysis based on the comparison of sperm and blastocyst DMR-associated genes revealed highly substantial gene enrichment between the two methylomes as paternal age increased. A total of 218 genes were identified with significant and directional DMRs, 61 genes were hypermethylated in both sources, 167 genes were hypomethylated, and 10 genes exhibited both hypo- and hypermethylated DMRs. Interestingly, significant enrichment of neurodevelopmental genes linked with autism spectrum disorder, schizophrenia, and bipolar disorder was observed in both APA sperm and blastocyst DMRs. This suggests that the changes in the sperm DNA methylation observed in APA are not randomly distributed within the genome, but may occur in imprinted genes involved in brain development, and persists through offspring by escaping widespread epigenetic reprogramming [72]. Therefore, we speculate that (1) age-related changes in DNA methylation may affect mutation rates in certain regions and (2) imprinted genes may be involved in mediating APA effects.

### Effect of Advanced Maternal Age on Offspring

#### Effects on Prenatal Outcomes

##### Maternal

Maternal age has been associated with a variety of prenatal outcomes. Two prospective epidemiological studies of all reproductive women in Denmark during 1978–1992 and Norway from 2009–2013 found that the risk of miscarriage, defined as the risk of loss between 6 and 20 weeks, was lowest among women aged 25–29 years (10%), increased rapidly after 30 years old, and reached 53% among women aged 45 and older [73, 74]. Both studies obtained data from female birth records, allowing better control of confounding factors such as reproductive history and calendar period. Furthermore, chromosomal abnormalities were linked to maternal age after a miscarriage (one or more times). Recently, karyotype analysis of 406 fetal tissues collected after spontaneous abortion in Germany from 2010 to 2014 revealed that the likelihood of chromosomal abnormality in a miscarriage increased by 6.4% every year. Notably, the increased likelihood of chromosomal abnormalities after miscarriage follows a *J*-shaped curve with maternal age [75]. Finally, a study which was conducted on singleton pregnancies in the USA involving 23,831 women, compared fetal death rates in mothers over 40 years old (*n* = 3953) to women under 35 years; and found that pregnancies with the former were more likely to result in fetal death [76]. Thus, clinical results suggests that maternal age influences prenatal outcomes.

##### Paternal

APA has been associated with some poor prenatal outcomes. In a recent U.S. study, an analysis of the combined 2011–2013 and 2013–2015 National Survey of Family Growth found paternal age may increase the odds of spontaneous miscarriage, independent of specific factors, including demographics, pregnancy intention, and maternal age [77]. Second, fetal deaths have also been correlated with paternal age. Researchers in Denmark conducted a prospective study of 23,821 pregnant women and discovered that the risk of fetal death was elated to paternal age. After accounting for maternal variables, the risk of fetal death was higher in males of 50 years or older than that in males < 50 [78]. An analysis of all Italian-born populations from 1990 to 1998 (*n* = 4,830,742) found that paternal age increases the preterm birth risk. Moreover, the risk increased more rapidly with paternal age for extremely preterm births than for moderately preterm births [79]. Overall, epidemiological evidence suggests that paternal age affects prenatal outcomes. However, the above results can only describe associations, not a direct effect, we believe that further understanding of the mechanisms underlying prenatal outcomes in APA is needed.

#### Effects on Emotion and Recognition in Offspring

Currently, the results of the studies based on human behavior are possibility contradictory, all of the findings can only demonstrate association and not causation [4-6]. However, a useful tool to investigate safety and risks of delayed parenthood is the use of the mouse model. The mouse could provide several advantages: (1) a defined genetic background; (2) the wide availability of validated and reliable behavioral, anatomical, and functional phenotyping methods; (3) shortener-generational intervals [80]. Therefore, we use the mouse model to understand the effects of parental age on offspring phenotype and the biological mechanisms underlying the occurrence of diseases in the descendants.

##### Maternal

The present studies suggested that (AMA) may affect offspring’s neurocognitive and emotional development [7, 81]. We summarized the present research (Table 2). Though there were inconsistent results, such as Tarin [82] and Mao [83] in the Morris water maze test, the discrepancy could result from the difference in the germline of the experimental animals and in the selected age of APA. In summary, all behavioral tests agree on that AMA caused anxiety-like behavior, social interaction, and learning and memory deficits in offspring rats.

**Table 2:** Effect of advanced maternal age on emotions and cognition of mouse offspring

Study	Strain	APA sire age	Behavioral examination findings	Experimental characteristics	Year
Tarin, J.J	C57BL/6JIco female*CBA/JIco male	12 months	Motor activity↓ Avoidance learning↓	The offspring of advanced women had lower step-through latencies in passive avoidance behavioral tests, but there were no changes in escape latencies five daily sessions in a Morris water maze test	2003 [82]
Sandra Lerch	C57BL/6N	8 months	Anxiety↑ Sociability↓	Sociability appears to be gender specific in offspring of normal maternal age. It was, however, absent in the offspring of aged mothers	2015 [84]
Sampino, S	Swiss Albino mice	15–18 months	Anxiety↑ Ultrasound vocalization (USV) activity ↑	These offspring were delivered by cesarean section, and fostered after birth by 3-month-old dams	2017 [85]
Mao, W.J	C57BL/6 J	8 months	Spatial learning ↓ Avoidance learning↓	They established mice model of the first natural pregnancy to avoid the confounders of potential adaptations from previous pregnancies Young adult offspring from aged mothers exhibited reduced VDR expression during fetoplacental development, which might play an important role	2018 [83]
Han, W	Sprague–Dawley rats	12 months	Sociability↓ Anxiety↑ Memory ↓	The effects of delayed motherhood on cognitive and emotional development in offspring are caused by CREB-dependent expression of BDNF	2018 [86]
Li, D	C57BL/6	8 months	Spatial learning ↓ Memory ↓ Anxiety↑	The significant impact of maternal vitamin D supplementation on the cognitive function of offspring	2021 [87]

Earlier research suggested that female mice exhibit different maternal behaviors at different ages, and that these changes may affect the behavior of their offspring [84]. The offspring in Sampino’s study, however, were all nursed by young foster mothers after delivery and were conceived by either young or old females. They discovered that the postnatal environment provided by young foster mothers did not reverse the effects of AMA, demonstrating that the brain programming induced by AMA is already established at birth [85]. Molecular biology also confirmed the effects on behavioral performance in offspring by the AMA. Gene expression in the hippocampus was altered in male offspring from AMA mice compared with young maternal (YMA) pregnant mice by microarrays. Several genes, including transcription factors Arc, Egr1, Fos, and Fkbp5, have differential mRNA expression and are involved in regulation of anxiety-related behaviors in rodents (these are important for synaptic plasticity and connectivity in the hippocampus). Furthermore, several genes involved in the heat shock response and unfolded protein response, such as Manf, Xbp1, Atf3, and heat shock proteins (i.e., Hspa1a, Hsp90b1, Hspa5, Hspe1, Hspb1, and Hsph1), were upregulated in hippocampi of male offspring conceived by old dams, this suggested that oxidative processes leading to neuronal aging may initiate earlier in the brain of offspring conceived by old females [85]. Thus, we consider that the effect of maternal age is more apparent in prenatal outcomes.

##### Paternal

There is abundant evidence that the paternal germline is a main source of de novo mutations in the human population, and more genetic information is altered in male sperm with increased paternal age. In many cases, age-related factors may alter genomic information carried by sperm through epigenetic reprogramming, leading to abnormal phenotypes and increased disease predisposition in offspring [88]. We summarize relevant animal studies of APA on offspring’s mood and cognition (Table 3). Paternal age is associated with psychiatric disease-related phenotypes in offspring (e.g.: impaired cognition, increased anxiety-like behavior and reduced sociability), this is due to changes in genetic information (e.g.: DNA methylation), generational and/or intergenerational effects may occur.

**Table 3:** Influence of advanced paternal age on emotions and cognition of mouse offspring

Study	Strain	APA sire age	Behavioral examination findings	Experimental characteristics	Year
M Aurous	Wistar rats	23 months	Learning capacity↓	There were gender differences in learning capacity	1983 [89]
Garcı´a-Palomares et al	C57BL/6JIco	25–30 months	Spontaneous motor↓ Avoidance learning↓	The authors set an APA “dose.” Delaying fatherhood in F_0_ mice until the age of 30 months has negative effects on postnatal development and behavioral traits of F_1_ offspring	2009 [90]
Rebecca G. Smith et al	C57BL/6 J	10 months	Social behaviors ↓ Exploratory ↓	This is the first examined the effect of older paternal age on social and non-social behavior in mice The authors only examined one strain of male mice	2009 [91]
Claire J. Foldi et al	C57BL/6 J	12–18 months	Anxiety↑ (female) Exploration↑(female) Learned helplessness↓ (female)	There were gender differences in behavioral phenotypes It may be useful to assess the effects of APA ‘dose’ in the present study	2010 [92]
S Sampino et al	Swiss albino mice	15 months	Ultrasound vocalization (USV) activity ↑ Sociability↓ Grooming activity↑ Anxiety ↑	This is the first model of transgenerational effects have been analyzed	2014 [88]
M. Janecka et al	C57BL/6 J	10–12 months	Juvenile social play↓ Adult social interaction↓	The authors assessed sociability in adult generation 0 male breeders and found that these social deficits were not present in the paternal Further confirmed that the de novo origins in the offspring generation and have revealed a sexually dimorphic developmental trajectory for social behavior	2015 [93]
Kaichi Yoshizaki et al	WT (C57BL6/JCrj)/Sey/ + mutant	12 months	Anxiety ↑ Locomotion ↑	Some phenotypes could be induced in combination of a genetic risk and an environmental risk	2016 [94]
Claire J. Foldi et al	C57BL/6 J	12–15 months 24 months	Anxiety: 24 months↑ Exploration: 24 months↓	Increasing paternal age was associated with an increase in severity of an anxiogenic phenotype in their adult offspring	2019 [95]
Wen-Long Zhao et al	C57BL/6 J	12–18 months	Anxiety↑ Sociability↓ Locomotion↓	Paternal aging could cause aberrant behaviors in intergenerational and transgenerational offspring	2020 [96]
Kaichi Yoshizaki et al	C57BL/6 J	> 12 months	Vocal communication↓	Further revealed that the molecular mechanisms of transgenerational epigenetics	2021 [71]

Molecular genetic study has identified a possible intergenerational epigenetic mechanism between paternal age and offspring phenotypes with psychiatric disorders. The authors discovered that paternal aging can result in infant vocal communication deficits. Using sperm DNA from young and aged mice, a targeted whole-genome methylome analysis revealed that older mice have more hypomethylated regions which have an enrichment of binding motifs for the RE1-silencing transcription factor (REST) also known as the neuron-restricted silencer factor (NRSF). However, REST/NRSF target genes were significantly upregulated in the developing brain of offspring derived from fathers of old age. Furthermore, abnormal behavior was observed in the offspring of young mice given a DNA demethylation drug. Thus, paternal aging may result in leaky expression of REST/NRSF target genes that have been hypomethylated within sperm cells, inducing premature neurogenesis and resulting in abnormalities in neuronal activities and brain structures, which may cause behavioral phenotypes associated with neurodevelopmental disorders [71].

**Fig. 2:**
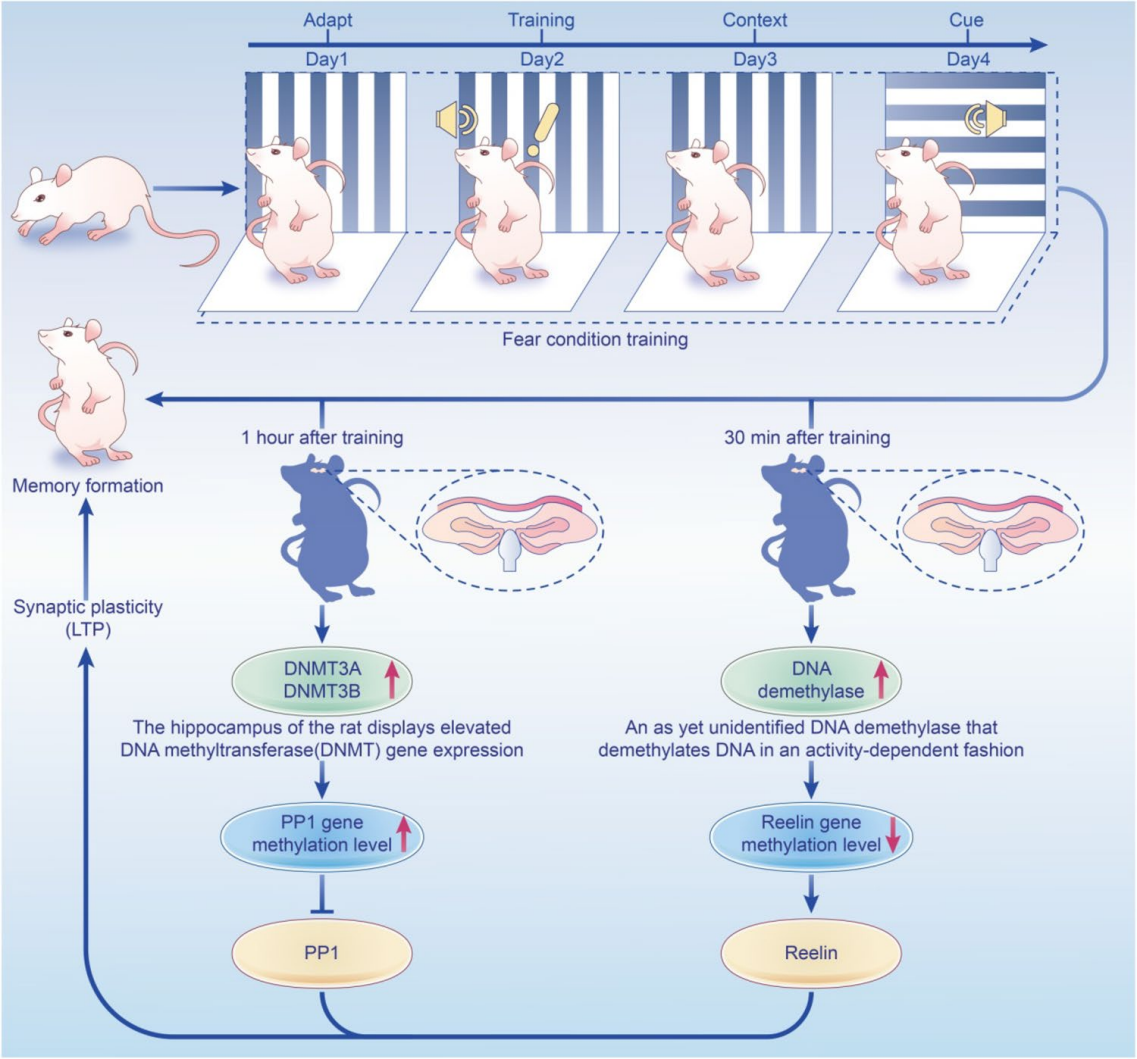
The adult nervous system regulates the methylation of DNA in a dynamic manner. After contextual fear conditioning training, the hippocampus of the rat displays elevated DNA methyltransferase (DNMT) gene expression, resulting in rapid methylation and transcriptional silencing of the memory suppressor gene PP1. Furthermore, demethylation of the synaptic plasticity gene Reelin increases, as does transcriptional activation. Inhibiting DNMTs, on the other hand, can prevent memory formation and increased PP1 methylation, leading to abnormal gene transcription during memory consolidation. As a result, dynamic regulation of DNA methylation in specialized contexts is an essential step in memory formation [98]

In addition to exploring the intergenerational epigenetic molecular mechanism, Kaichi Yoshizaki et al. also proposed whether DNA hypomethylation of REST/NRSF-binding motifs in sperm affects the REST/NRSF target genes expression in the embryonic brain and subsequent behavioral abnormalities. The underlying mechanism may underlie the scenario of paternal exposure to environmental stimulus in rats. We believe that environmental factors (e.g., stress in life) may change the expression of genes associated with abnormal behavior in offspring from older parents. Recently, Luo et al. explored the effects of parental age, environmental stimuli, and gender differences on offspring’s mood, learning, and memory through chronic unpredictable mild stress (CUMS), which is considered an inevitable stress during individual development. Their results found impaired fear memory and spatial memory in female offspring with advanced parental age [97]. In addition, the results of Miller Ca and Sweatt JD found that after experiencing context dependent fear conditioning (the training procedure causes some stress to the animals), DNMT gene and PP1 gene methylation levels increased in the hippocampus of the animals, while Reelin methylation levels decreased. Inhibition of DNMTs in the hippocampus leads to impaired scene dependent fear memory (Fig. 2) [98]. The above studies suggest that DNA methylation plays a key role in the formation and maintenance of memory in a special environment. Therefore, it is an interesting question whether changes in the environment cause changes in cognition and emotion-related gene methylation is the cause of the abnormal phenotypes of the elderly offspring. The role of environmental factors in APA effects is unclear. But given that correlation between gene and environment may lead to APA effects, future studies should not only refine the relevant experimental design, but also interpret the results of animal studies cautiously. Only then can we better judge the risk of developing an associated psychiatric disorder in the population by detecting behavioral phenotypes.

## Summary and Future Prospects

**Fig. 3:**
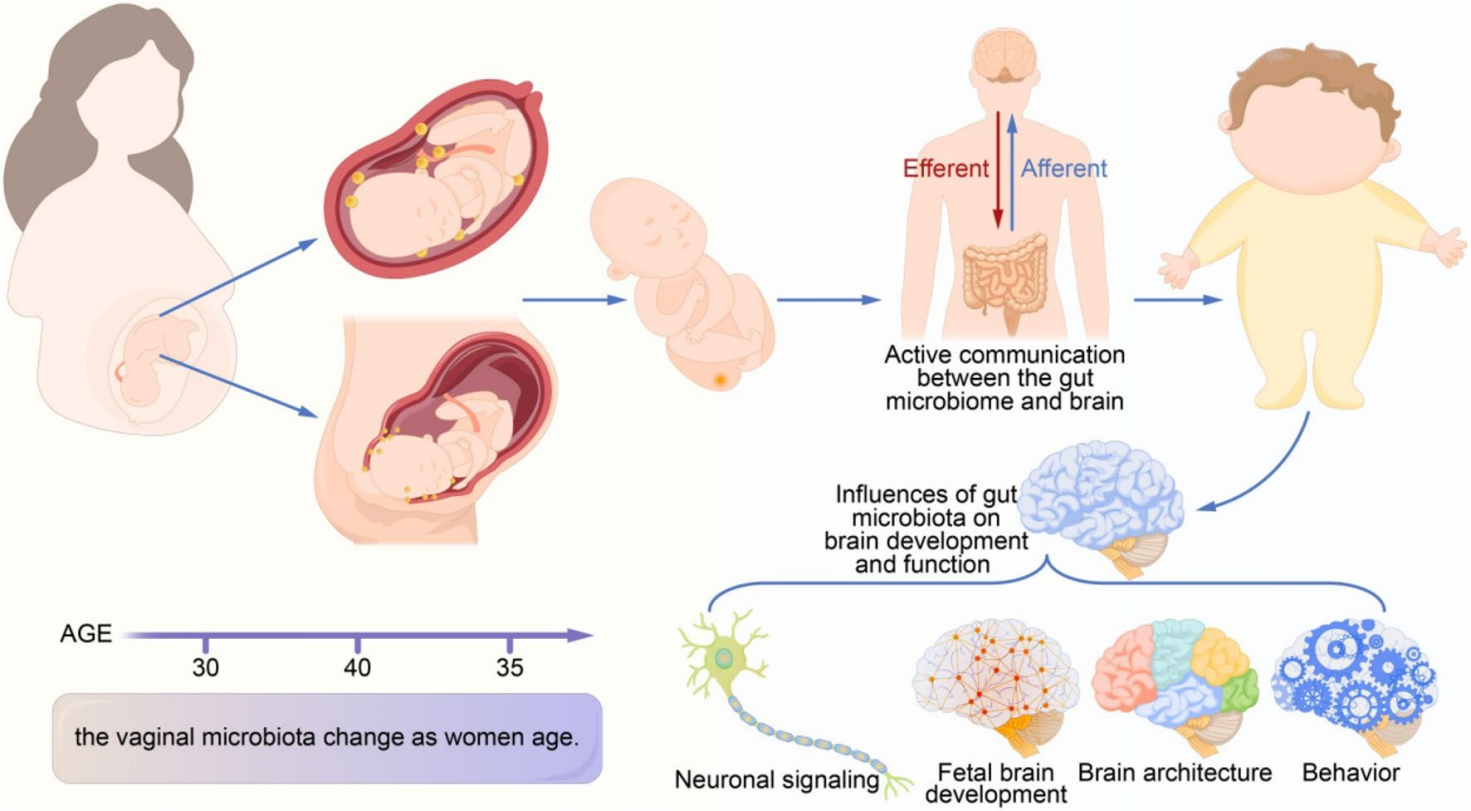
With increasing age, the flora in women may change and affect the gut flora colonization as well as the development of the fetus. The current studies found that the sex hormone may change and affect the vaginal microbiota change in women with increasing age [24, 103]. A recent study firstly suggests that the dysregulation in vaginal of women with AMA [104]. However, the amniotic fluid plays a more important role than the vaginal microbiota in the microbiota colonization of the neonatal gut [105]. Unfortunately, we were not able to retrieve studies on maternal age and amniotic fluid biota, which will be one of the future focuses of our laboratory team. However, early microbiome colonization has been linked to infant brain development. Animal studies have shown that changes in the maternal microbiome or early postnatal microbiome caused by aseptic feeding, antibiotic treatment, or other environmental factors may lead to abnormalities in brain immunity, blood–brain barrier permeability, brain structure, and neural circuits regulating the generation, identity, and maturation of neurons in the offspring. These effects may persist into adulthood and predispose to long-term behavioral deficits, underscoring the importance of maintaining microbiome balance during a critical window in neurodevelopment [106]. As a result, we will investigate the relationship between different reproductive ages, the in vivo flora of pregnant women, and the future colonization of newborns' intestinal flora and deeply explore the relationship between specific bacteria and adverse offspring behavioral phenotypes in woman with AMA

Advanced parental age is correlated with significantly increased offspring’s genetic risk. However, the exact age at which the risk occurs and the severity of the risk is unknown. From these studies, we found the following:

- Observed alterations in the telomere and epigenetic profiles of germ cells are related to advanced parental age. Therefore, we consider the DNA methylation clock and telomeres to be the candidates to predict the biological age of female reproduction. But, the ovarian function begins to decline in women after age 30, compared with men who can be produced germ cells during the their life cycle. We will be pay more attention to the application of telomere and epigenetic profiles on maternal with the prediction for reproduction ability (specifically, epigenetic profiles) [48, 99]. We consider the DNA methylation clock (a precise biological age marker was developed by researchers and named Horvath’s epigenetic clock [100]) to be the most promising candidates to predict the biological age of female reproduction. In addition, as for the application of telomeres in female with the prediction for reproduction ability, although a lot of studies have reported the role of telomeres in female fertility prediction. we cannot predict or determine how much telomere shortening occurs in each pregnancy, which may impact future predictions of pregnancy success rates for women (it is possible that some women were unable to conceive after one pregnancy) [101].
- The increased risk of psychiatric disorder in the offspring of parents with advanced parental age may occur in the form of abnormal behavioral phenotypes.
- The health of children born by elderly mothers may be developmentally programmed before birth. In other words, the abnormal characteristics in offspring from old mother was inherent before birth and exists in life span. From the epigenetic map, the effect of maternal age on offspring may be limited by a “threshold” from mother. This threshold could depend on the mother’s life style, health, and living standard. The threshold protects the DNA methylation to be maintained and acquired in imprinted genes, which allow these imprinted genes have normal methylation. We think this is the "”mother’s love” that exists in the genetic information, which can protect the health of the offspring against the abnormal development of the offspring.
- The effects of APA on offspring may occur more after the offspring is born, and exert intergenerational/transgenerational genetic effects.
- Do AMA have no effect on the development of their offspring after birth? We think that there’s another type of genetic information that might play a role—microbiome. We speculate that the female flora may change with age and affect the colonization of the fetal gut flora, thereby affecting critical processes in the development of organ systems including the fetal brain (Fig. 3).
- The impact of environmental factors on cognitive and emotional development of older offspring will be an interesting direction. Recently, we have focused on the impact of socio status on the cognitive and emotional development of the offspring from advanced parental. At this stage, there was a good animal model to test. In 2017, Zhou et al. showed that they proved the winner effect and established animal models and reflected the social hierarchy phenomenon in animals [102]. We made an interesting hypothesize that the offspring of advanced parents with higher social class have more active behavioral and emotional traits than the offspring of advanced parents with lower social class. If this hypothesis is true, we believe that parents’ social class advantages can be preserved in genetic information and transmitted to offspring and influenced for the cognitive and emotional development on offspring.

## Data Availability

Data availability is not applicable to this article as no new data were created or analyzed in this study.
